# Preparation of Porous Carbon Nanofiber Electrodes Derived from 6FDA-Durene/PVDF Blends and Their Electrochemical Properties

**DOI:** 10.3390/polym13050720

**Published:** 2021-02-26

**Authors:** Do Geun Lee, Byeong Chul Lee, Kyung-Hye Jung

**Affiliations:** School of Advanced Materials and Chemical Engineering, Daegu Catholic University, Gyeongsan, Gyeongbuk 38430, Korea; kms911120@gmail.com (D.G.L.); bclee@live.co.kr (B.C.L.)

**Keywords:** carbon nanofibers, supercapacitors, 6FDA–durene, PVDF, precursor blend, coin cells

## Abstract

Highly porous carbon electrodes for supercapacitors with high energy storage performance were prepared by using a new precursor blend of aromatic polyimide (PI) and polyvinylidene fluoride (PVDF). Supercapacitor electrodes were prepared through the electrospinning and thermal treatment of the precursor blends of aromatic PI and PVDF. Microstructures of the carbonized PI/PVDF nanofibers were studied using Raman spectroscopy. Nitrogen adsorption/desorption measurements confirmed their high surface area and porosity, which is critical for supercapacitor performance. Energy storage performance was investigated and carbonized PI/PVDF showed a high specific capacitance of 283 F/g at 10 mV/s (37% higher than that of PI) and an energy density of 11.3 Wh/kg at 0.5 A/g (27% higher than that of PI) with high cycling stability.

## 1. Introduction

For energy storage devices such as lithium-ion batteries (LiBs) or supercapacitors, electrode materials are one of the most critical components in determining energy storage performance. For LiBs, high porosity of the electrode materials provides a large surface area for Faradaic reaction and short distances for charge diffusion [1]. It is also well-known that supercapacitors store electrical energy in the interface between an electrolyte and an electrode, and that the utilization of porous electrode materials can enlarge their interfacial area [2].

Carbon nanofibers (CNFs) have gained considerable attention as promising electrode materials due to their superior electrochemical properties, chemical and thermal stability, and ready synthesis [3,4,5]. They are obtained in the form of nonwoven mats, allowing them to be used as an electrode directly without adding any binders or additives. One of the typical methods for synthesizing CNFs is the thermal treatment of precursor polymer nanofibers fabricated by electrospinning. Thermal treatment includes stabilization, which is to treat electrospun nanofibers under air at around 250–400 °C, and carbonization, which is to treat stabilized nanofibers under inert gas at over 800 °C. CNFs can be also produced by catalytic chemical vapor deposition (CVD), with CVD-grown CNFs being known to have high contents of crystalline carbon, resulting in high electrical conductivity [4,5].

To produce CNFs, polyacrylonitrile (PAN), polybenzimidazole (PBI), aromatic polyimides (PIs), and polyvinylidene fluoride (PVDF) have typically been reported as precursor polymers [6,7,8,9,10,11,12,13,14]. Their copolymers and polymer blends have been also studied as precursors in order to improve the electrochemical properties of the resultant CNFs. For copolymer precursors, vinyl imidazole, itaconic acid, and acrylic acid have been reported as comonomers of PAN, and CNFs derived from these copolymers show higher electrochemical performance compared to those from PAN only [15,16,17]. It is also known that using polymer blends as carbon precursors is effective in producing porous CNFs with excellent energy storage performance. These polymer blends generally consist of precursor polymers and sacrificial polymers such as polymethyl methacrylate (PMMA), polylactic acid (PLA), or polyvinylpyrrolidone (PVP) [18,19,20,21]. Sacrificial polymers show lower thermal stability and can be decomposed during stabilization, resulting in a large surface area and high porosity of the resultant CNFs. Li et al. produced porous carbon electrodes for LiBs using PAN/PLA blends, showing a large surface area and good electrochemical performance [19]. In addition to using sacrificial components to prepare polymer blends, two polymers that can both act as carbon precursors are blended to produce CNF electrodes. For example, aromatic PI/PBI blends were reported as effective CNF precursors that exhibit excellent surface properties and energy storage performance [22,23].

Due to the energy storage mechanism, the surface area and porosity of electrode materials are some of the most critical factors in improving the number of active sites. Among precursor polymers, hexafluoroisopropylidene diphthalic anhydride (6FDA)-based PIs have a high free volume due to the presence of bulky trifluoromethyl (CF_3_–) groups, which can facilitate polymer chain rearrangement during stabilization [24]. Moreover, highly porous carbon can be fabricated through the thermal treatment of 6FDA-based PIs since the decomposition of CF_3_– groups can generate a large number of pores. It was reported that CNFs derived from 6FDA-based PIs exhibit a large surface area and high porosity compared to those derived from PAN or PBI with sacrificial polymers [10,11].

PVDF has been investigated for use in the field of energy storage and harvesting, such as in solid electrolytes and separators [25,26,27,28,29]. It has been also studied as a blend component since it can provide a blend matrix with mechanical and dimensional stability. PI/PVDF blends were reported as proton exchange membranes for fuel cell, separators for LiBs, and gas separation membranes [30,31,32]. It was also reported that aromatic PI can act as a nucleating agent for PVDF crystallization, which can make them compatible blends [33]. Moreover, PVDF is one of the carbon precursor polymers; however, the carbonization of PVDF requires a catalyst or additional chemical treatment [12,13,14].

In this study, the strategy for producing highly porous CNF electrodes with high energy storage performance is to use 6FDA-based PI/PVDF blends. 6FDA-based PI can improve the thermal stability of PVDF, eliminating the need for additional compounds or treatment, while the decomposition of PVDF that is less thermally stable than PI can create additional pores on the surface of the resultant CNFs. CNFs were successfully produced through the electrospinning and thermal treatment of PI/PVDF blends. Nitrogen adsorption/desorption behavior was investigated to study the surface area and porosity of CNFs. To measure supercapacitor performance, coin cells were assembled with CNF electrodes derived from PI and PI/PVDF as symmetrical electrodes with an aqueous electrolyte. Optimization of the blend ratio of PI and PVDF was conducted by measuring the electrochemical properties of CNFs derived from PI/PVDF with different blend ratios.

## 2. Materials and Methods

To synthesize PI, 4,4-(hexafluoroisopropylidene) diphthalic anhydride (>99%, 6FDA), 2,3,5,6-tetramethyl-1,3-phenyldiamine (99%, durene-diamine), anhydrous dimethyl acetamide (99.8%), triethylamine (99%), and ethylene diamine were purchased from Tokyo Chemical Industry Co., Tokyo, Japan, and acetic anhydride was purchased from Sigma-Aldrich, St. Louis, MO, USA. 1-Methyl-2-pyrrolidinone (99%, NMP) obtained from Tokyo Chemical Industry Co. was used as a co-solvent for 6FDA–durene and PVDF (Sigma-Aldrich, MW = 180,000). To evaluate the electrochemical properties, coin cells were assembled using CR2032 coin blanks (MTI Corporation, Richmond, CA, USA) with glass microfiber filters (separator, GE Healthcare Life, Marlborough, MA, USA) as a separator and potassium hydroxide (Sigma-Aldrich, 99.99%) as an electrolyte.

6FDA–durene was synthesized according to the literature procedures [34]. The molecular weight of the synthesized 6FDA–durene was 75,000 g/mol, determined using a gel permeation chromatography (GPC) system equipped with Alliance e2695 (Waters Corporation, Milford, MA, USA) and tetrahydrofuran (THF) as a solvent. 6FDA–durene/PVDF (90:10) dissolved in NMP was chosen as a precursor blend based on the preliminary study for optimizing a blend ratio of PI and PVDF. The 16 wt% 6FDA–durene/PVDF dissolved in NMP was electrospun, and 6FDA–durene nanofibers were also prepared for comparison using an electrospinning/spray system (ESR100, eS-robot^®^, NanoNC, Seoul, Korea). There was a significant difference between the molecular weight of the synthesized PI (75,000) and the purchased PVDF (180,000). Thus, to have appropriate electrospinnability and comparable fiber diameters, the concentration of PI/PVDF and PI solutions were set as 16% and 20%, respectively. The applied voltage was controlled in the range of 9 to 15 kV depending on environmental conditions such as relative humidity in the electrospinning chamber. Electrospun nanofibers were stabilized at 350 °C in a box furnace (FX-05, Daihan Scientific Co., Wonju, Korea) for 2 h under air, and carbonized at 800 °C in a tube furnace (FT-860.3, Daihan Scientific) for 30 min under nitrogen.

Chemical structures of the electrospun nanofibers were investigated by attenuated total reflectance Fourier transform infrared (ATR-FTIR, 4100, Jasco, Easton, MD, USA). Thermal stability was measured using thermogravimetric analysis (TGA, DTA-60, DSC-60, Shimadzu, Tokyo, Japan) by heating up to 800 °C at a heating rate of 10 °C min^−1^. The surface morphology of the electrospun and carbonized PI/PVDF nanofibers was observed using scanning electron microscopy (SEM) with an SU8220 (Hitachi, Tokyo, Japan) after sputter coating with gold. Raman spectroscopy was recorded to study the carbon microstructures of carbonized PI/PVDF using an inVia reflex (Renishaw, UK) with a 780 nm laser. Surface area and porosity of CNFs were obtained using nitrogen adsorption/desorption measurement on an Autosorb-iQ and a Quadrasorb SI (Quantachrome, Boynton Beach, FL, USA). Specific surface area was calculated from nitrogen adsorption isotherms using the Brunauer–Emmett–Teller (BET) equation, and pore size distribution was calculated using density functional theory (DFT) methods.

Supercapacitor performance was investigated through cyclic voltammetry (CV), cycling performance, and galvanostatic charge–discharge using a WBCS3000S (Wonatech, Seoul, Korea). They were measured by assembling symmetric coin cells using two identical CNF electrodes without the use of binders or additives. Glass fiber membrane and 6.0 M potassium hydroxide (KOH) were introduced as a separator and an electrolyte, respectively. The specific capacitance (*C*_sp_, F/g) was measured from CV measurements using Equation (1) as follows:

(1) $$C_{sp}=\frac{4}{m}\frac{I}{\nu }$$

where *m* is the mass of both electrodes (g), *I* the discharge current (A), and *ν* the scan rate (V/s). Capacitance retention after 5000 cycles under the voltage range of −0.5 to 0.5 V and a scan rate of 100 mV/s was measured to confirm the cycling stability.

From a galvanostatic charge–discharge test under a voltage range of 1.0 to 0 V and a discharge current density range of 0.5 to 5 A/g, energy density (*E*, Wh kg^−1^) and power density (*P*, W kg^−1^) were calculated using Equations (2) and (3), respectively, as follows:

(2) $$E=\frac{I_{d}t_{d}V}{2m}$$

(3) $$P=\frac{E}{t_{d}}$$

where *I_d_* is the discharge current (A), *t_d_* the discharge time (s), and *V* the voltage window (V). Electrochemical impedance spectra (EIS) were measured over the frequency range from 100 kHz to 0.1 Hz using a 0.01 V amplitude on a PEC-L01 (Peccek, Japan).

## 3. Results and Discussion

### 3.1. Characterization of CNFs Derived from 6FDA–Durene/PVDF

**Figure 1:**
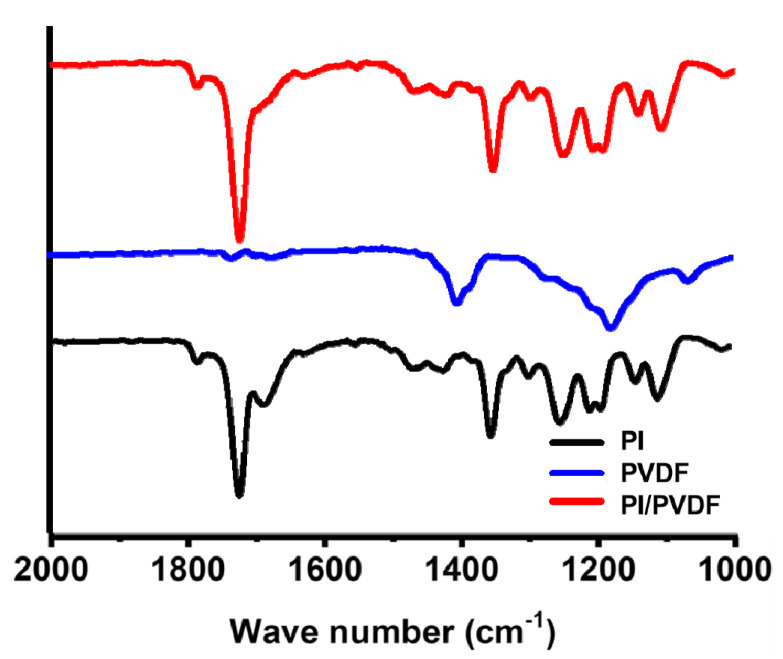
FTIR (Fourier transform infrared) spectra of PI (aromatic polyimide), PVDF (polyvinylidene fluoride), and PI/PVDF.

FTIR analysis of the synthesized and electrospun 6FDA–durene were carried out, as shown in Figure 1. Successful imidization is confirmed by distinctive bands at 1723, 1786, and 1354 cm^−1^ for the C=O symmetric stretch, C=O asymmetric stretch, and C–N stretch of imide, respectively. A peak for the C–F stretch is also found at 1207 cm^−1^. The chemical structure of the electrospun PVDF nanofibers was also observed, and an adsorption peak at 1178 cm^−1^ assigned to the symmetrical stretching of –CF_2_ groups is shown [35].

Synthesized 6FDA–durene and PVDF (90:10) were blended and then electrospun to produce precursor blend nanofibers. Figure 1 also shows that peaks indicating 6FDA–durene are clearly seen while those for PVDF do not appear in the FTIR spectra of the PI/PVDF blend, which may result from a low content of PVDF (10%).

**Figure 2:**
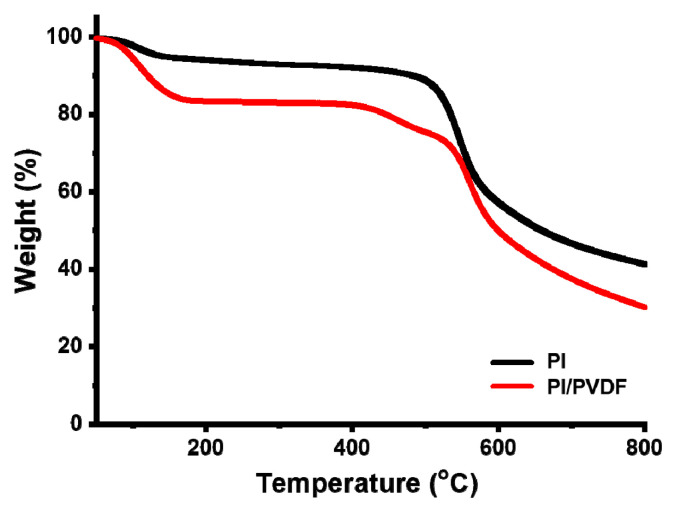
Thermogravimetric analysis of PI and PI/PVDF.

Figure 2 shows the thermal stability of electrospun PI and PI/PVDF nanofibers. PI nanofibers show thermal stability up to 550 °C, at which the carbonyl groups in the imide start to break [36]. It is also shown that a residue of 46% of the sample weight remains at 800 °C. For PI/PVDF blend nanofibers, a slight weight loss occurs at 390 °C, which is higher than the degradation temperature of PVDF (350 °C) [37,38]. It was reported that carbonization of PVDF only required a catalyst or additional chemical treatment [12,13,14]. Blending with thermally stable polymers such as 6FDA-based PIs can enable PVDF to withstand high temperatures and to convert to carbon without additional compounds or treatment. Nevertheless, PVDF is less thermally stable than PI and it can be decomposed during thermal treatments (i.e., stabilization and carbonization), resulting in the creation of pores on the CNF surfaces.

**Figure 3:**
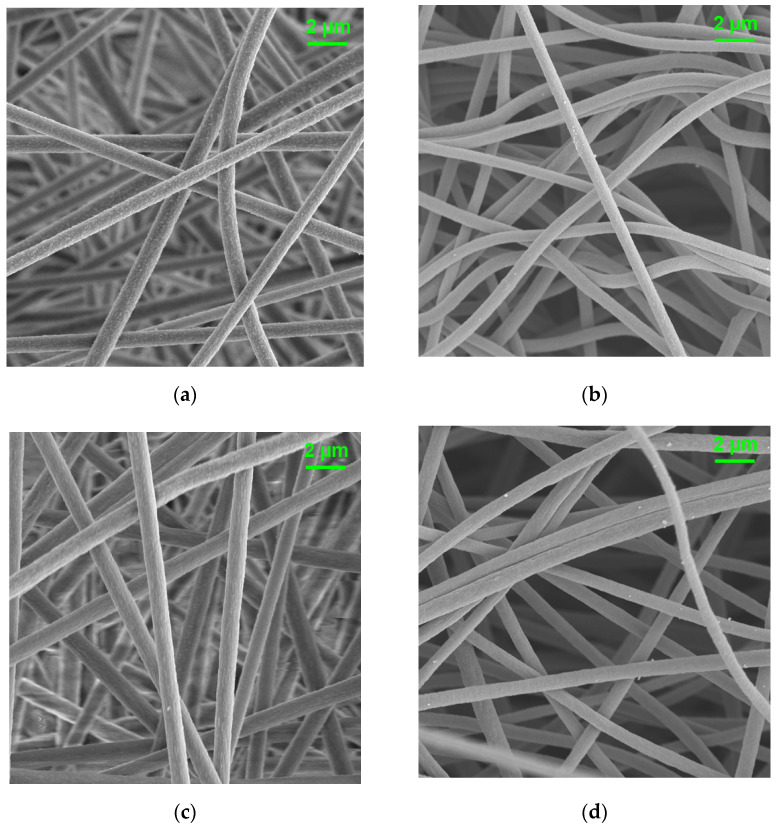
Surface morphology of (**a**) as-spun PI, (**b**) carbonized PI, (**c**) as-spun PI/PVDF, and (**d**) carbonized PI/PVDF nanofibers.

The nanofibrous morphology of electrospun and carbonized PI and PI/PVDF was observed via SEM, as shown in Figure 3. Figure 3a shows that the electrospinning of 6FDA–durene successfully forms nanofibers with fiber diameters of 770 ± 20 nm. After carbonization, a decrease in fiber diameters to 740 ± 30 nm is found, as shown in Figure 3b. In Figure 3c,d, it is observed that fiber diameters are increased by adding 10% PVDF to PI, resulting in fiber diameters of 825 ± 50 nm for electrospun PI/PVDF and 770 ± 10 nm for carbonized PI/PVDF. It can also be seen that nanofibrous structures of both PI and PI/PVDF are well preserved after the thermal treatment of stabilization at 350 °C and carbonization at 800 °C due to their high thermal stability.

**Figure 4:**
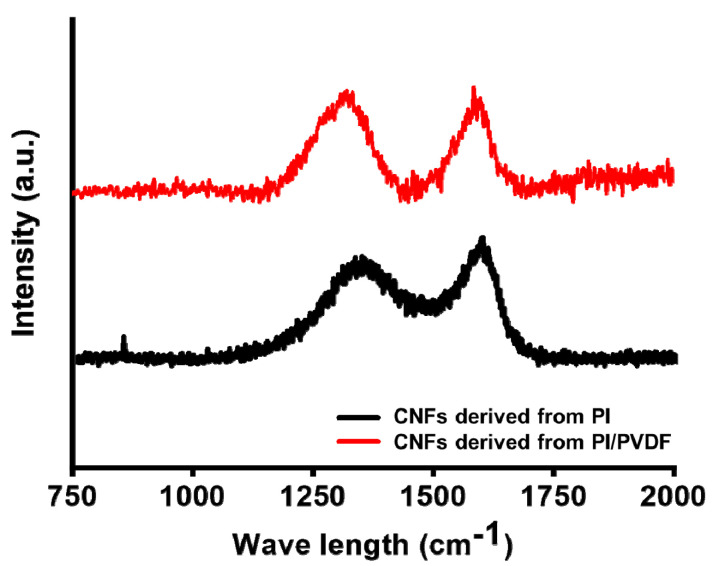
Raman spectra of CNFs (carbon nanofibers) derived from PI and PI/PVDF.

Electrospun PI and PI/PVDF were stabilized and carbonized to produce CNFs, and their carbon microstructures were observed by Raman spectroscopy, as shown in Figure 4. Both carbonized PI and PI/PVDF nanofibers show two significant peaks around 1340 cm^−1^ (D-band) and 1590 cm^−1^ (G-band). It is known that D-band and G-band represent the presence of disordered carbon and oriented graphitic carbon, respectively [39]. The relative intensity ratio of the D-band to the G-band (*I*_D_/*I*_G_) was also measured, and PI/PVDF-derived CNFs show a lower ID/IG ratio of 1.7 than that of PI-derived CNFs (1.9), which means that more ordered carbon is formed in PI/PVDF-derived CNFs.

6FDA-based PIs are considered as an outstanding candidate for CNF precursors due to their high free volume caused by the presence of bulky trifluoromethyl (CF_3_–) groups [40]. During oxidative stabilization, polymer chains are rearranged to convert to cyclic and ladder-like structure and high free volume can enhance their mobility [41]. The addition of PVDF can facilitate polymer chain rearrangement in PIs, resulting in lower *I*_D_/*I*_G_ ratio. Abeykoon et al. reported the fabrication of CNFs using PBI/PI blends, and CNFs-derived PBI/PI blends contained more ordered carbon than those from PBI only [23].

### 3.2. Surface Properties of CNFs Derived from 6FDA–Durene/PVDF

**Figure 5:**
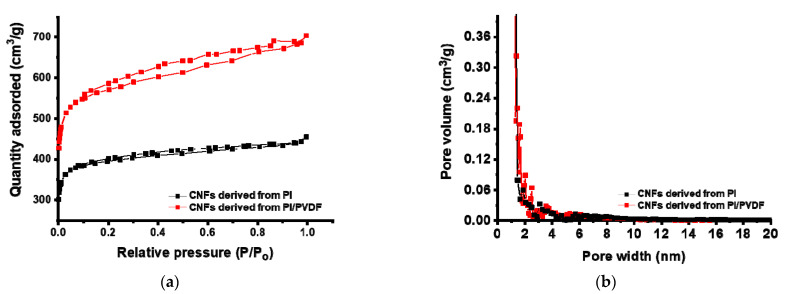
Nitrogen adsorption/desorption isotherms (**a**) and pore size distribution (**b**) of CNFs.

CNFs were characterized by nitrogen adsorption/desorption analyses as shown in Figure 5a for carbonized PI and PI/PVDF nanofibers. Carbonized PI/PVDF adsorbs 700 cm^3^/g of nitrogen at 0.99 of the relative pressure, which is a 55% increase over carbonized PI. It can also be seen that CNFs derived from PI/PVDF present a high SSA of 1559 cm^2^/g, which is significantly higher than those from PI only (1210 cm^2^/g), as shown in Table 1.

Figure 5b shows the DFT-calculated distribution of pore volumes in CNFs, which are summarized in Table 1. It can be seen that the presence of 10% PVDF causes an 18% increase in TPV, with a 14% increase in micro-pore volume and a 42% increase in meso-pore volume. Due to the lower thermal stability of PVDF compared to PI, PVDF seems to be decomposed during thermal treatments, which can create pores on the resultant CNF surface.

Among energy storage devices, supercapacitors store energy at relatively high rates compared to batteries due to their simple energy storage mechanism of ion adsorption/desorption at the electrode/electrolyte interface [42]. Thus, many researchers have mainly focused on the enhancement of the surface area and porosity of electrode materials to obtain expanded ion accumulation. One of the most common approaches is to use pore-generating agents which exhibit low thermal stability. It was reported that porous CNFs were successfully prepared by using PMMA as a pore-generating agent and that adding 5% PMMA to PAN led to 11.1% and 30.8% increases in SSA and TPV, respectively [18]. PLA was also used as a pore-generating agent due to its low thermal stability, and CNFs derived from PBI/PLA exhibited 29.2% and 34.3% larger SSA and TPV, respectively, compared to those from PBI only [20].

6FDA-based PIs are considered to be excellent precursor polymers for highly porous carbon [10,11,40]. During thermal treatment, the decomposition of bulky fluorinated substituents in the 6FDA unit can generate pores, resulting in a high surface area and high porosity in the resultant CNFs. It was reported that the surface properties of CNFs derived from 6FDA–durene were further improved by surface treatment of the precursor nanofibers prior to carbonization, leading to a 12.8% increase in SSA and a 10.3 % increase in TPV [43].

### 3.3. Electrochemical Performance of CNFs Derived from 6FDA–Durene/PVDF

Coin cells were assembled with free standing carbonized PI and PI/PVDF nanofibers without using additives or binders, and the supercapacitor performances of the CNF electrodes were studied using CV and galvanostatic charge–discharge testing. To optimize the blend ratio, CNFs derived from PI/PVDF with different ratios of 95:5, 90:10 and 85:15 were prepared, and their electrochemical properties were measured.

**Figure 6:**
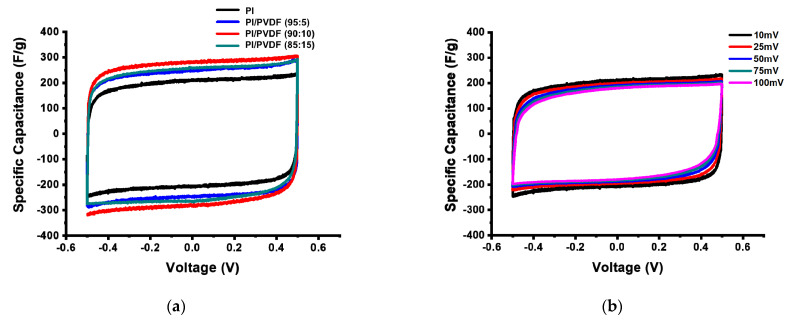

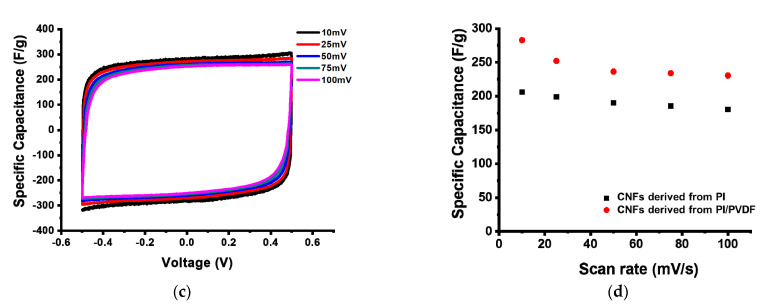
Cyclic voltammograms of (**a**) CNFs derived from PI/PVDF with different PVDF contents, (**b**) CNFs derived from PI, and (**c**) CNFs derived from PI/PVDF (90:10) with different scan rates and (**d**) specific capacitances over scan rates.

Figure 6a shows the CVs of CNFs derived from PI/PVDF with different blending ratios conducted under a scan rate of 10 mV/s. It is clearly seen that all four CNF electrodes show typical rectangular shapes for electrical double layer capacitors under a potential range of –0.5 to 0.5 V, and that CNFs derived from PI/PVDF exhibit high capacitances compared to those from PI only (206 F/g). Moreover, it can be found that CNFs derived from PI/PVDF (90:10) show the highest capacitance of 283 F/g.

Recently, several 6FDA-based PIs have been reported as precursor polymers of CNF electrodes, showing excellent energy storage performance. Porous CNF electrodes prepared by using 6FDA–durene only without the use of any sacrificial compounds showed a specific capacitance of 139 F/g at 10 mV/s with an ionic liquid electrolyte, which is higher than those from PAN or PBI with the pore-generating materials [10]. Introducing PVDF can further improve energy storage performance by enlarging the surface area and increasing porosity, which are apparently higher than in PAN or PBI-based CNFs. It was also reported that CNFs derived from crosslinked 6FDA–durene exhibited a high specific capacitance of 301 F/g compared to that of non-treated ones (205 F/g) with KOH [43]. CNFs derived from 6FDA-2-2′-bis(trifluoromethyl) benzidine (TFMB) were also reported, and steam-activated CNFs showed a specific capacitance of 292 F/g at 10 mV/s with KOH [11]. It seems that the addition of PVDF has a comparable effect on improving surface porosity and energy storage performance with pre- (surface crosslinking of PI nanofibers before carbonization) or post-treatment (steam activation of carbonized PI nanofibers).

Figure 6b,c show the CVs of CNFs derived from PI and PVDF/PI (90:10) with different scan rates, respectively. At high scan rates, the interaction between the electrolyte ions and the electrode surfaces and pores is limited, resulting in low specific capacitances [44]. Itt is shown that PI/PVDF-derived CNF electrodes exhibit significantly higher capacitances than PI-derived ones for all scan rates, as shown in Figure 6d.

**Figure 7:**
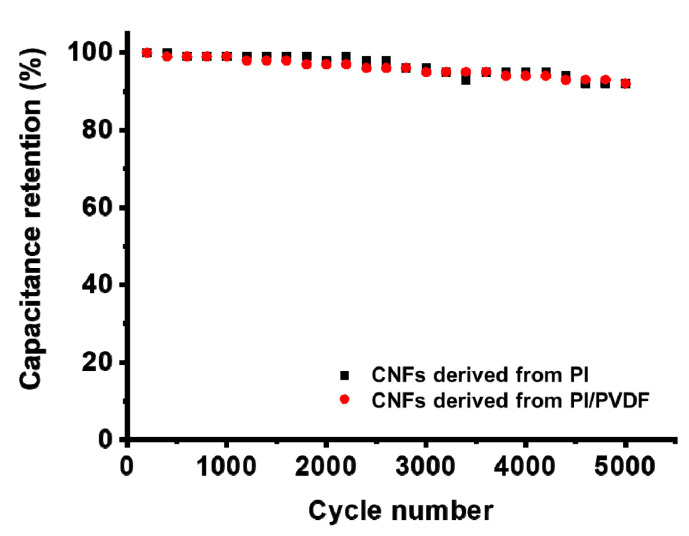
Cyclic performance at 100 mV/s of CNFs derived from PI and PI/PVDF.

One of the great advantages of supercapacitors is their long cycle life, since they store charges physically in the electrochemical double layers formed at the electrode surface without irreversible redox reactions [45]. In Figure 7, the cycling stability of CNFs derived from PI and PI/PVDF was evaluated by measuring the specific capacitance at 100 mV/s for every 100 cycles, and both exhibit a high capacitance retention of 92% after 5000 cycles. A high degree of reversibility demonstrates the stable cyclic performance of these CNF electrodes.

**Figure 8:**
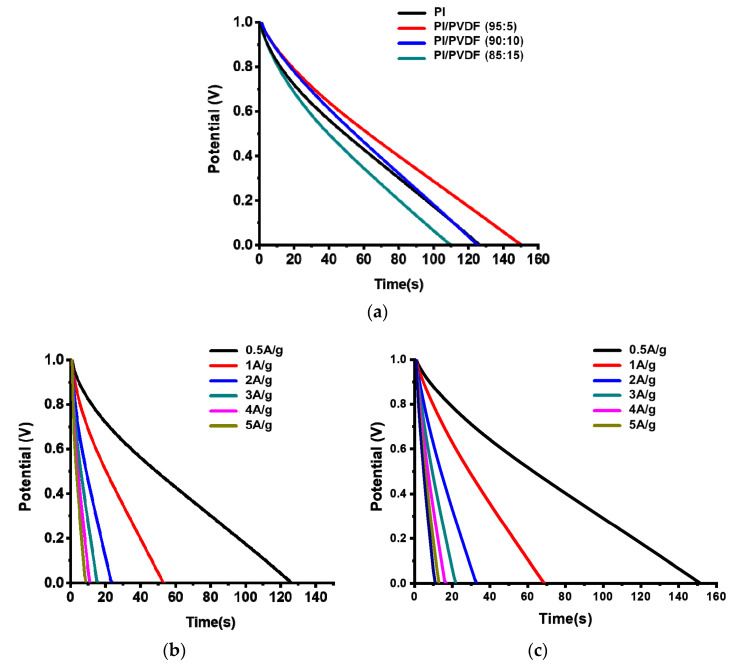
Galvanostatic discharge curves of (**a**) CNFs derived from PI/PVDF with different PVDF contents, (**b**) CNFs derived from PI, and (**c**) CNFs derived from PI/PVDF (90:10) with different current densities.

Galvanostatic charge–discharge was conducted to study energy storage performance of CNF electrodes. Optimization of a blend ratio of PI and PVDF was also done with a charge–discharge test under a current density of 0.5 A/g and a potential range of 1.0–0 V, as shown in Figure 8a. Among CNFs, CNFs derived from PI/PVDF (90:10) show the longest discharge time, resulting in the highest energy density of 11.3 Wh/kg, which is consistent with the CV results in Figure 6a.

Figure 8b,c show the discharge curves of CNFs derived from PI and PI/PVDF, respectively, over a current density range of 0.5–5 A/g. It is apparent that CNFs derived from PI/PVDF have higher energy densities due to the longer discharge times than those from PI only (8.9 Wh/kg at 0.5 A/g).

Polymer blends have been studied to produce highly porous CNF electrodes with good energy storage performance. Previously, PBI/PI blends were chosen as precursors for supercapacitor electrodes since both PBI and PI were well-known carbon precursor polymers. It was reported that PBI/Matrimid^®^ blends were good precursor candidates for CNF electrodes, showing superior surface and electrochemical properties [22]. During thermal treatment, Matrimid^®^ was partially damaged, while its blends with PBI were successfully converted to carbon. The addition of PBI improved the thermal stability of the blend matrix through hydrogen bonding between PBI and PI. Matrimid^®^, which is thermally unstable compared to PBI, and was partially decomposed during thermal treatment, which resulted in the creation of pores in the CNF surface and thus improving the surface area and porosity. As a result, the addition of 25% Matrimid^®^ led to 48% and 54% increases in specific capacitance and energy density, respectively. 6FDA-based PI was also used to prepare PBI/PI blends for CNF precursors [23]. It was found that CNFs derived from PBI/PI (70:30) blends exhibited 88% and 76% larger specific capacitance and energy density, respectively, than those from PBI only.

Recently, biopolymers such as cellulose acetate or lignin have gained attention as carbon precursors since they are widely available and are low-cost renewable sources. Blends of PAN and these biopolymers were studied as CNF precursors for high thermal stability and processability [46,47]. The resultant CNFs derived from PAN/cellulose acetate and PAN/lignin showed improved energy storage performance compared to those from PAN only due to their higher surface area and porosity.

**Figure 9:**
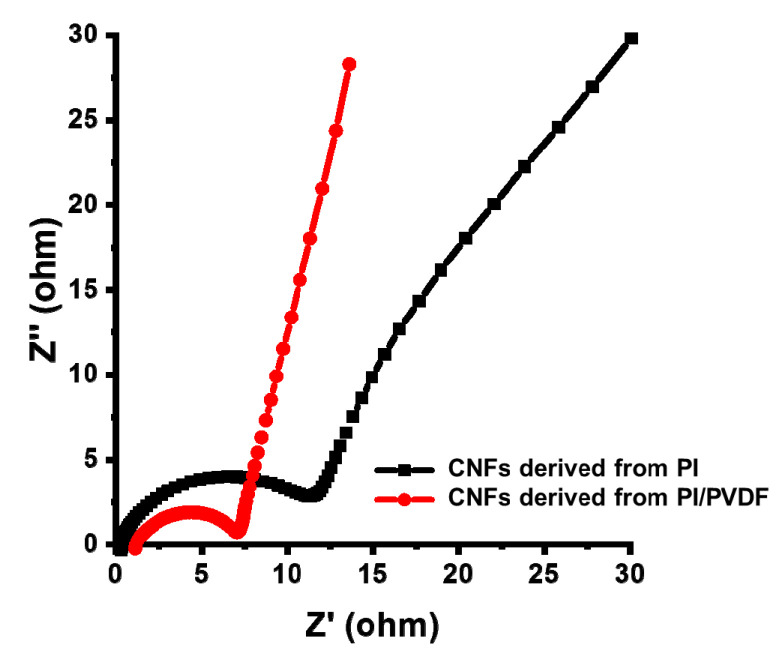
EIS (electrochemical impedance spectra) of CNF electrodes derived from PI and PI/PVDF.

The electrochemical impedance of the CNF electrodes was measured as shown in Figure 9. The intercept of the semi-circle with the real axis represents the sum of the resistance of the electrolyte, the intrinsic resistance of the electrode material, and the interfacial resistance between the electrode and the current collectors [48,49]. Therefore, the size of the semi-circle at high frequency is determined by the resistance of the electrode materials [50]. The charge–transfer resistance (Rct) of the CNFs derived from PI/PVDF, half of the value of the real semicircle in the high-frequency region, is 3.25 Ω/cm, which is lower than those from PI (6.40 Ω/cm). Highly porous CNF electrodes enable fast electrolyte ion accumulation and diffusion onto the electrode surface, resulting in low resistance and superior energy storage performance.

The large surface area of the electrode materials leads to short distances for charge diffusion, low resistance, and good cyclic stability in supercapacitors [42]. It can be confirmed that successful preparation of highly porous CNF electrodes using PI/PVDF as precursor blends results in superior energy storage performance such as high specific capacitances, energy densities, and electric conductivity. In addition to the surface properties of electrode materials, it is known that supercapacitor performance is governed by the type of electrolyte used, which determines the voltage window. Energy density is proportional to the square of the operating voltage, and thus using ionic liquid electrolyte with a high working voltage can improve energy densities [51]. It was found that CNFs derived from 6FDA–durene exhibited a high energy density of 63.4 Wh/kg (at 1 A/g) with an ionic liquid electrolyte [10]. Thus, it seems worthwhile to investigate the supercapacitor performance of porous CNF electrodes derived from PI/PVDF with an ionic liquid to obtain further improved performance.

Another approach to improving energy storage performance is to introduce pseudocapacitance, which involves reversible faradaic reactions. For pseudocapacitors, metal oxides or conductive polymers have been investigated as electrode materials. Moreover, metal oxide/carbon composite electrodes are widely studied [52,53,54,55]. It seems possible that these porous CNF electrodes derived from PI/PVDF can act as an excellent carbon matrix for composite electrodes. More study will be conducted to see their potential as electrode materials for other energy storage devices.

## 4. Conclusions

Highly porous CNFs were fabricated through the electrospinning and thermal treatment of precursor blends of 6FDA-based PI and PVDF. Thermally stable PI enabled successful conversion to carbon, while PVDF played a role in the creation of pores on CNF surfaces due to its relatively lower thermal stability. Nitrogen adsorption/desorptiinon data confirmed that the addition of PVDF to PI successfully developed a larger pore volume and a wider pore size distribution on the CNF surface. According to CV and galvanostatic charge–discharge testing, the best composition of PI/PVDF (90:10) was selected as a CNF precursor. CNFs derived from PI/PVDF showed a high specific capacitance of 283 F/g at 10 mV/s (37% higher than those from PI) and an energy density of 11.3 Wh/kg at 0.5 A/g (27% higher than those from PI) with high cycling stability, confirming the successful fabrication of highly porous CNF electrodes using a new precursor blend of PI and PVDF. Further improvement of supercapacitor performance was expected by fabricating composite electrodes with these porous CNF electrodes as a carbon matrix and using conductive polymers or metal oxides for pseudocapacitance.

## Figures and Tables

**Table 1:** Surface properties of CNFs derived from (a) PI and (b) PI/PVDF.

	SSA ^1^ (m^2^/g)	TPV ^2^ (cm^3^/g)	V_micro_^3^ (m^3^/g)	V_meso_^4^ (m^3^/g)
PI	1210	0.580	0.496	0.084
PI/PVDF	1559	0.684	0.565	0.119

^1^ SSA: specific surface area. ^2^ TPV: total pore volume. ^3^ V_micro_: micro-pore (<2 nm) volume. ^4^ V_meso_: meso-pore (2–50 nm) volume.

## Data Availability

The data presented in this study are available in this article.
